# Supplementary material for: Binding and structural basis of equine ACE2 to RBDs from SARS-CoV, SARS-CoV-2 and related coronaviruses
Source: Nat Commun. 2022 Jun 21;13:3547. doi: 10.1038/s41467-022-31276-6 (PMC9210341; doi:10.1038/s41467-022-31276-6)
Supplement: Supplementary file 1 — Supplementary Information [file 41467_2022_31276_MOESM1_ESM.pdf]

Supplementary Information

Binding and structural basis of equine ACE2 to RBDs from SARS-CoV, SARS-CoV-2 and related coronaviruses

Zepeng Xu[1,2,5], Xinrui Kang[1,3,5], Pu Han[1,5], Pei Du[1,5], Linjie Li[1,4], Anqi Zheng[1,4], Chuxia Deng[2], Jianxun Qi[1,4], Xin Zhao[1], Qihui Wang[1,4,*], Kefang Liu[1,*], George F. Gao[1]

[5] These authors contributed equally.

* Correspondence: wangqihui@im.ac.cn (Q.W.) and Liukf@im.ac.cn (K.L.)

Supplementary Figures

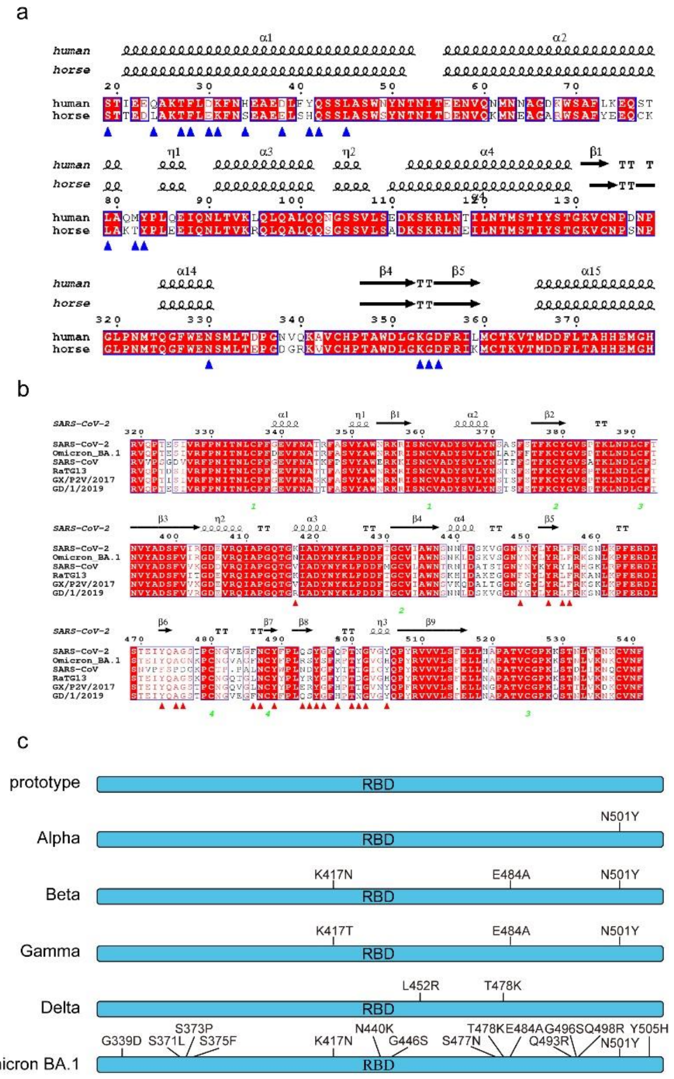

Supplementary Fig. 1 Structure-based sequence alignment of RBDs and ACE2 orthologs and schematic diagram of substitutions observed in SARS-CoV-2 VOC RBDs. a Structure based alignment of eqACE2 and hACE2. Residues contacting the RaTG13-RBD of hACE2 and eqACE2 are respectively labeled with blue triangles and red stars. Identical residues are highlighted white on a red background, and residues in red on a white background indicate a similarity score >0.7, considering physio-chemical properties. The alignment was performed by T-COFFEE and visualized by ESPript 3.0. **b** Alignment of SARS-CoV-2 PT, Omicron BA.1, SARS-CoV and related CoVs. Residues of SARS-COV-2 PT-RBD contacting with human ACE2 were labelled with red triangles. **c** Schematic diagram for substitutions in RBDs of the VOCs.

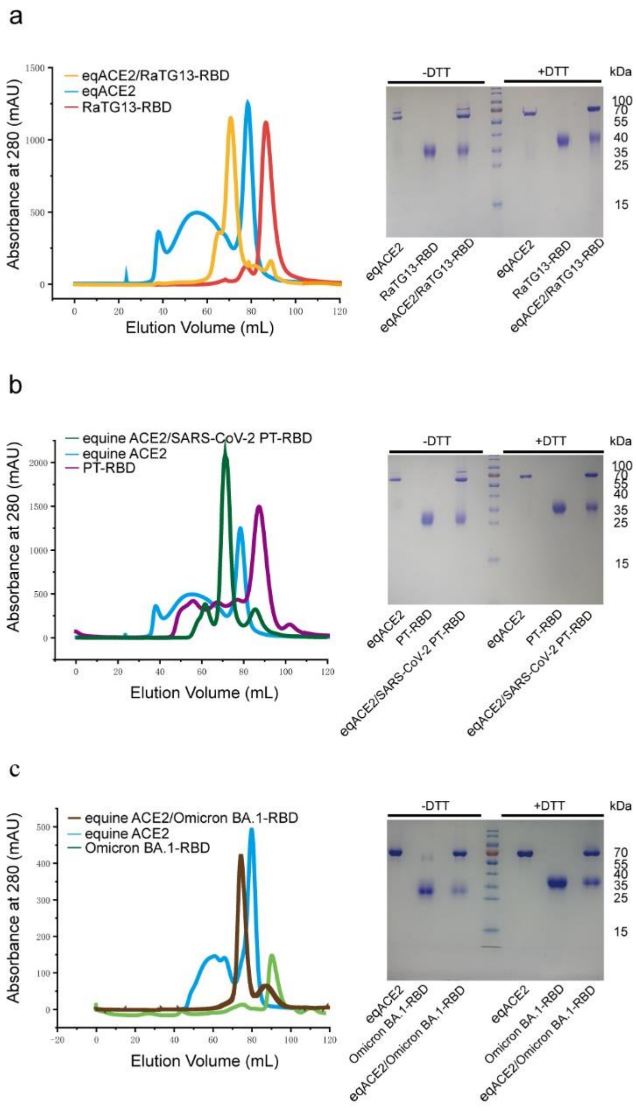

Supplementary Fig. 2 Protein preparation of the eqACE2/RaTG13-RBD, eqACE2/SARS-COV-2 PT-RBD and eqACE2/Omicron BA.1-RBD complexes. a-c Gel filtration profiles of eqACE2 (blue), RaTG13-RBD (red), SARS-COV-2 PT-RBD (purple), Omicron BA.1-RBD (light green), eqACE2/RaTG13-RBD complex (yellow) and eqACE2/SARS-COV-2 PT-RBD complex (dark green). Separation profiles of each pooled sample on SDS-PAGE are shown with and without DTT. Source data are provided as a Source Data file.

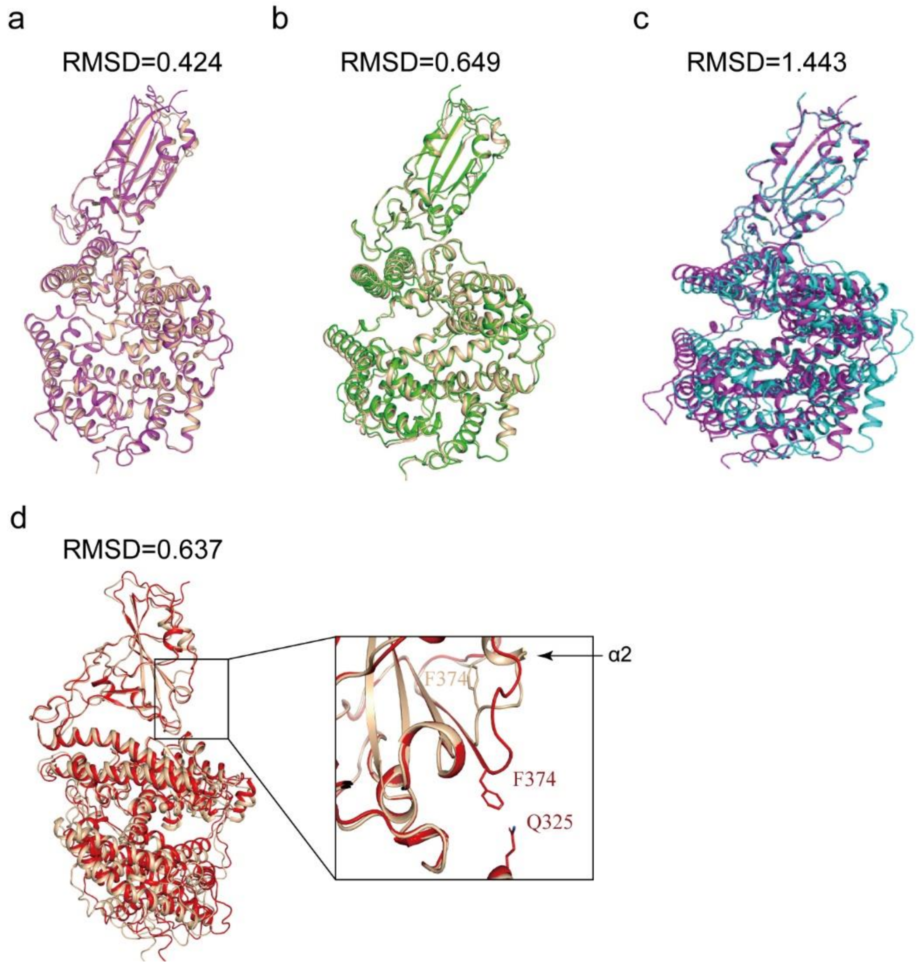

Supplementary Fig. 3 Multiple alignments of the relevant complexes and the stretched α2 helix of eqACE2/Omicron BA.1-RBD. a-d Alignment between the eqACE2/RaTG13-RBD (purple) and eqACE2/SARS-COV-2 PT-RBD (wheat) (**a**), eqACE2/SARS-COV-2 PT-RBD and hACE2/SARS-COV-2 PT-RBD (green) (**b**), eqACE2/RaTG13-RBD (purple) and hACE2/RaTG13-RBD (cyan) complexes (**c**). RaTG13-RBD binds to eqACE2 at a different angle from its binding to hACE2. (**d**) Alignment between the eqACE2/SARS-COV-2 PT-RBD (wheat) and eqACE2/Omicron BA.1-RBD (red). The α2 helix, F374 of RBDs in both complexes and Q325 of eqACE2 in eqACE2/Omicron BA.1-RBD are labeled. RMSD is labeled above the alignment.

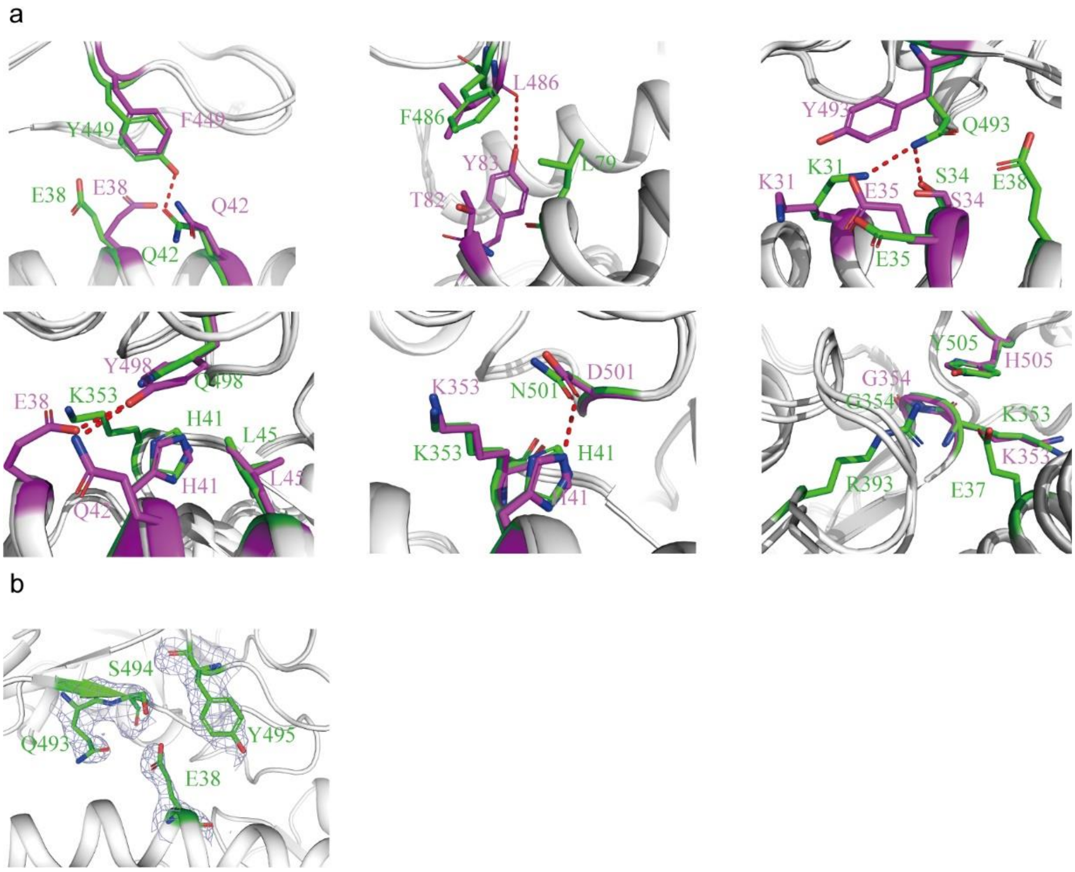

Supplementary Fig. 4 Structural comparison of RaTG13-/SARS-COV-2 PT-RBD in complex with eqACE2 as well as RaTG13-/SARS-COV-2 PT-RBD complexed with hACE2. a Substituted residues of RaTG13-RBD (purple) and SARS-COV-2 PT-RBD (green) and their interacting residues on eqACE2 are displayed as sticks and colored in purple and green, respectively. **b** The electron density of E38 and its main interacting residues of eqACE2/SARS-COV-2 PT-RBD.

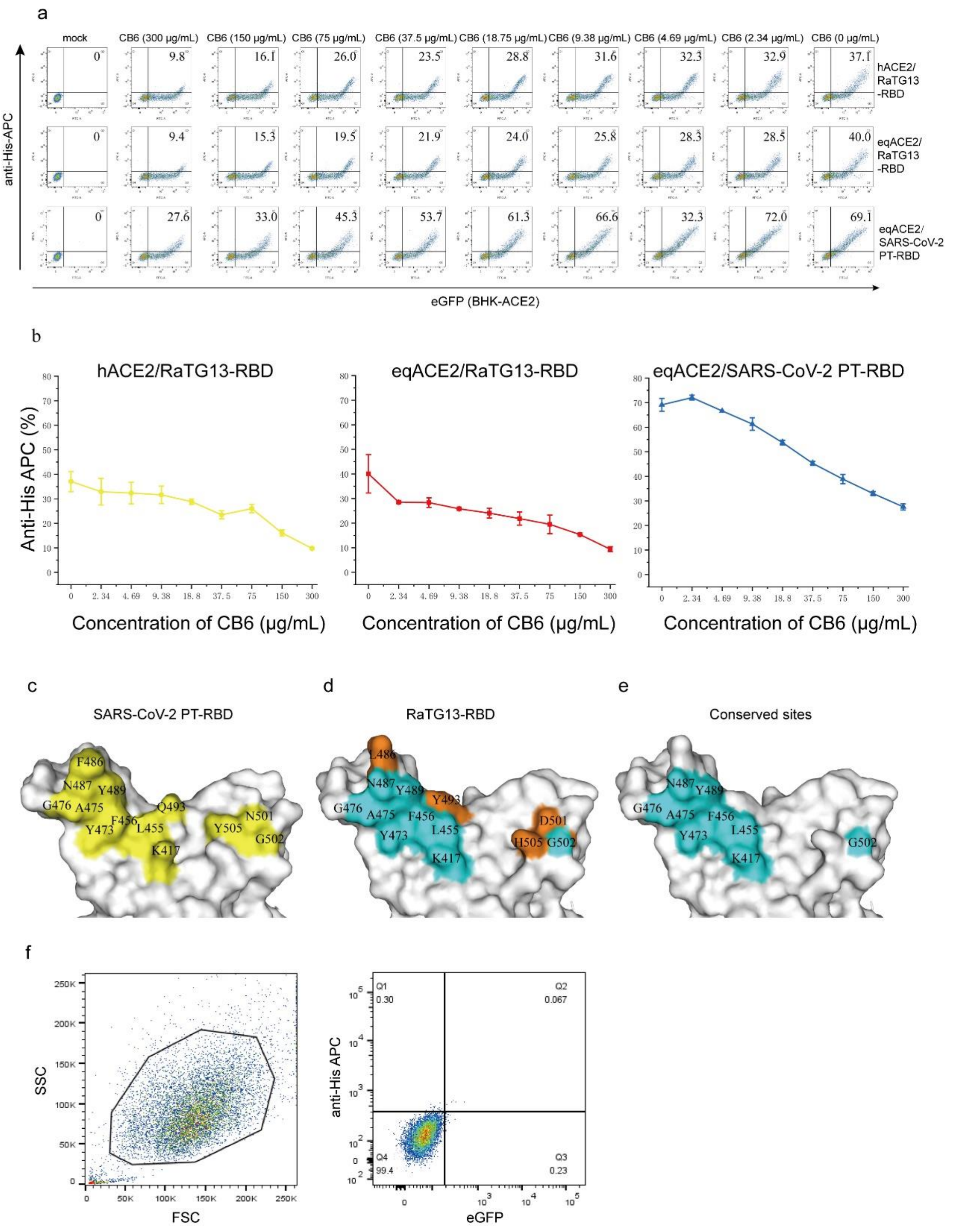

Supplementary Fig. 5 Cross-reactive immunity of SARS-CoV-2 to RaTG13. a One set of representative results of flow cytometry of CB6 cross-binding. The frequency of RBD binding-positive cells among the ACE2-eGFP-positive cells is labelled in the upright corner. **b** Blocking efficiency of CB6 against binding of RBDs and ACE2

orthologs. The data derives from two biologically independent experiments and are presented as mean ± SD. Source data are provided as a Source Data file. **c** Overlapping residues of the SARS-COV-2 PT-RBD binding to hACE2 and CB6. **d** Counterpart residues of the RaTG13-RBD; substitutions are colored in orange. **e** Conserved sites among the overlapping interface between the SARS-COV-2 PT-RBD and RaTG13-RBD. **f** Gating strategy for flow cytometric analysis of the binding between ACE2s and SARS-CoV-2 PT-RBD or RaTG13-RBD. Live cells are gated first, blank cells (not transfected by ACE2-eGFP) are used to determine criteria for eGFP and anti-His APC positive.

Supplementary Table

Supplementary Table 1 Crystallographic data collection and refinement statistics.

	eqACE2/RaTG13-RBD	eqACE2/SARS-CoV-2-RBD	eqACE2/SARS-CoV-2-Omicron BA.1RBD
**Data collection**			
Space group	$P\ 2_1$	$I\ 4_1\ 2\ 2$	$P\ 3_1\ 2\ 1$
Cell dimensions			
a, b, c (Å)	64.78, 122.84, 68.80	195.57, 195.57, 149.77	113.98, 113.98, 152.74
α, β, γ (°)	90.00, 92.42, 90.00	90.00, 90.00, 90.00	90.00, 90.00, 120.00
Wavelength (Å)	0.97852	0.97918	0.97918
Resolution (Å)	50.00-2.60 (2.69-2.60)	31.39-2.56 (2.63-2.56)	50.00-2.86 (2.96-2.86)
Unique reflections	32977 (3285)	46770 (3434)	27326 (2682)
R_{merge}	0.171 (1.032)	0.172 (2.286)	0.160 (1.252)
$I / \sigma I$	10.5 (1.8)	17.8 (2.0)	13.1 (1.4)
Completeness (%)	99.9 (100.0)	99.9 (100.0)	99.7 (100.0)
Redundancy	6.3 (6.4)	26.8 (26.6)	7.5 (6.6)
**Refinement**			
Resolution (Å)	46.16-2.60 (2.69-2.60)	19.21-2.56 (2.65-2.56)	41.45-2.86 (2.95-2.86)
No. of reflections	30722 (2132)	46567 (4598)	25611 (1258)
R_{work} / R_{free}	0.1846/0.2453	0.1934/0.2265	0.2298/0.2626
No. of atoms			
Protein	6424	6373	6416
Ligand/ion	1	1	21
Water	166	71	0
B-factor			
Protein	43.2	67.0	54.7
Ligand/ion	41.0	125.0	86.2
Water	36.9	57.7	-
R.m.s.deviations			
Bond length (Å)	0.004	0.004	0.003
Bond angles (°)	0.690	0.730	0.581
Ramachandran analysis			
Most favored (%)	96.32	97.44	96.19
Allowed (%)	3.55	2.43	3.30
Disallowed (%)	0.13	0.13	0.51

*Values in parentheses are for highest-resolution shell.

Supplementary Table 2 Amino acid residues comparison of RaTG13-RBD and SARS-COV-2 PT-RBD interacting with eqACE2

RaTG13-RBD/SARS-COV-2 PT-RBD	eqACE2 (RaTG13-RBD)	eqACE2 (SARS-COV-2 PT-RBD)
K417	E30 (7, **1**)	E30 (6, **1**)
F449/Y449	E38 (6), Q42 (3)	E38 (4), Q42 (8, **1**)
Y453	S34 (2)	S34 (2)
L455	E30 (2), K31 (2), S34 (1),	E30 (2), K31 (3), S34 (2)
F456	T27 (4), E30 (6), K31 (4)	T27 (7), E30 (7), K31 (4)
Y473	T27 (1)	T27 (1)
A475	S19 (3, **1**), L24 (4)	S19 (1), L24 (5), T27 (2)
G476	S19 (3), L24 (2)	S19 (1), L24 (2)
L486/F486	T82 (2), Y83 (2, **1**)	L79 (3)
N487	L24 (7), Y83 (5, **1**)	L24 (13), Y83 (5, **1**)
Y489	L24 (1), T27 (2), F28 (8), K31 (6), Y83 (1, **1**)	L24 (1), T27 (8), F28 (8), K31 (4), Y83 (1)
Y493/Q493	K31 (6), S34 (3), E35 (7)	K31 (4, **1**), S34 (10, **1**), E35 (1). E38 (3)
R494/S494		E38 (8, **2**)
Y495		E38 (2)
G496	E38 (3), K353 (6, **1**)	E38 (1), K353 (6)
Y498/Q498	E38 (6, **1**), H41 (7), Q42 (4, **1**), L45 (4),	H41 (8), L45 (4), K353 (1)
T500	L45 (1), N330 (9), D355 (9, **1**), R357 (5)	L45 (1), N330 (8), D355 (9, **2**), R357 (7)
D501/N501	H41 (4, **1**), K353 (6)	H41 (5), K353 (9)
G502	K353 (4, **1**), G354 (6), D355 (1)	K353 (4, **1**), G354 (7), D355 (2)
H505/Y505	K353 (23), G354 (4)	E37 (3), K353 (25), G354 (5), R393 (1)
Total	207, **11**	234, **10**

The numbers in parentheses of eqACE2 (RaTG13-RBD) and eqACE2 (SARS-COV-2 PT-RBD) residues represent the number of vdw contacts between the indicated residues with eqACE2. The numbers with underline suggest numbers of potential H-bonds between the pairs of residues. vdw contact was analyzed at a cutoff of 4.5 Å and H-bonds at a cutoff of 3.5 Å.

Supplementary Table 3 Amino acid residues comparison of hACE2 and eqACE2 interacting with RaTG13-RBD

eqACE2/hACE2	RaTG13-RBD (eqACE2)	RaTG13-RBD (hACE2)
S19	A475 (3, **1**), G476 (3)	S477 (2, **1**)
L24/Q24	A475 (4), G476 (2), N487 (7), Y489 (1)	A475 (3), G476 (4), N487 (12), Y489 (1)
T27	F456 (6), Y473 (1), A475 (1), Y489 (6)	F456 (11), Y473 (1), A475 (2), Y489 (4)
F28	Y489 (8)	Y489 (7)
E30/D30	K417 (7, **1**), L455 (2), F456 (6)	K417 (5), L455 (2), F456 (4)
K31	L455 (2), F456 (4), Y489 (6), Y493 (6)	L455 (2), F456 (4), Y489 (7), Y493 (11, **1**)
S34/H34	Y453 (2), L455 (1), Y493 (3)	Y453 (6), L455 (12)
E35	Y493 (7)	
E38/D38	F449 (6), G496 (3), Y498 (6, **1**)	F449 (8), G496 (1), Y498 (6, **1**)
H41/Y41	Y498 (7), D501 (4, **1**)	Y498 (11), T500 (8, **1**), D501 (8)
Q42	F449 (3), Y498 (4, **1**)	G446 (1), Y498 (5, **1**)
L45	Y498 (4), T500 (1)	Y498 (2), T500 (2)
L79		L486 (1)
T82/M82	L486 (2)	L486 (1)
Y83	L486 (2, **1**), N487 (5, **1**), Y489 (1, **1**)	N487 (10, **2**), Y489 (2, **1**)
N330	T500 (9)	T500 (6)
K353	G496 (6, **1**), D501 (6), G502 (4, **1**), H505 (23)	G496 (5), Y498 (2), D501 (10), G502 (5, **1**), H505 (20)
G354	G502 (6), H505 (4)	G502 (6), H505 (4)
D355	T500 (9, **1**), G502 (1)	T500 (7, **1**), G502 (1)
R357	T500 (5)	
Total	207, **11**	232, **10**

The numbers in parentheses of RaTG13-RBD (eqACE2) and RaTG13-RBD (hACE2) residues represent the number of vdw contacts between the indicated residues with RaTG13-RBD. The numbers with underline suggest numbers of potential H-bonds between the pairs of residues. vdw contact was analyzed at a cutoff of 4.5 Å and H-bonds at a cutoff of 3.5 Å.

Supplementary Table 4 Amino acid comparison of hACE2 and eqACE2 interacting with SARS-COV-2 PT-RBD

eqACE2/hACE2	SARS-COV-2 PT-RBD (eqACE2)	SARS-COV-2 PT-RBD (hACE2)
S19	A475 (1), G476 (1)	A475 (3, **1**), G476 (4)
L24/Q24	A475 (5), G476 (2), N487 (13), Y489 (1)	A475 (4), G476 (5), N487 (15, **1**)
T27	F456 (7), Y473 (1), A475 (2), Y489 (8)	F456 (5), Y473 (1), A475 (2), Y489 (7)
F28	Y489 (8)	Y489 (7)
E30/D30	K417 (6, **1**), L455 (2), F456 (7)	K417 (4, **1**), L455 (2), F456 (4)
K31	L455 (3), F456 (4), Y489 (4), Q493 (4, **1**)	L455 (2), F456 (5), E484 (1), Y489 (6), F490 (2), Q493 (3)
S34/H34	Y453 (2), L455 (2), Q493 (10, **1**)	Y453 (5, **1**), L455 (9), Q493 (6)
E35	Q493 (1)	Q493 (8)
E37	Y505 (3)	Y505 (7)
E38/D38	Y449 (4), Q493 (3), S494 (8, **2**), Y495 (2), G496 (1)	Y449 (9, **1**), G496 (5), Q498 (1)
H41/Y41	Q498 (8), N501 (5)	Q498 (8), T500 (7, **1**), N501 (8, **1**)
Q42	Y449 (8, **1**)	G446 (4, **1**), Y449 (4, **1**), Q498 (8, **3**)
L45	Q498 (4), T500 (1)	Q498 (3), T500 (1)
L79	F486 (3)	F486 (2)
T82/M82		F486 (9)
Y83	N487 (5, **1**), Y489 (1)	F486 (11), N487 (8, **1**), Y489 (1)
N330	T500 (8)	T500 (8)
K353	G496 (6), Q498 (1), N501 (9), G502 (4, **1**), Y505 (25)	G476 (7, **1**), N501 (11), G502 (4, **1**), Y505 (28)
G354	G502 (7), Y505 (5)	Y502 (7), Y505 (4)
D355	T500 (9, **2**), G502 (2)	T500 (8, **1**), G502 (1)
R357	T500 (7)	T500 (3)
R393	Y505 (1)	Y505 (1)
Total	234, **10**	288, **16**

The numbers in parentheses of SARS-COV-2 PT-RBD (eqACE2) and SARS-COV-2 PT-RBD (hACE2) residues represent the number of vdw contacts between the indicated residues with SARS-COV-2 PT-RBD. The numbers with underline suggest numbers of potential H-bonds

between the pairs of residues. vdw contact was analyzed at a cutoff of 4.5 Å and H-bonds at a cutoff of 3.5 Å.

Supplementary Table 5 Amino acid residues comparison of Omicron BA.1-RBD and SARS-COV-2 PT-RBD interacting with eqACE2

Omicron BA.1-RBD/ SARS-COV-2 PT-RBD	eqACE2 (Omicron BA.1-RBD)	eqACE2 (SARS-COV-2 PT-RBD)
F374	Q325 (4)	
R403	K353 (1)	
N417/K417		E30 (6, **1**)
Y449	E38 (9, **1**), Q42 (1, **1**)	E38 (4), Q42 (8, **1**)
Y453	S34 (2)	S34 (2)
L455	E30 (2), K31 (1)	E30 (2), K31 (3), S34 (2)
F456	T27 (5), E30 (4), K31 (3)	T27 (7), E30 (7), K31 (4)
Y473	T27 (1)	T27 (1)
A475	L24 (2), T27 (3)	S19 (1), L24 (5), T27 (2)
G476	L24 (4),	S19 (1), L24 (2)
N477/S477	S19 (5, **1**)	
F486	L79 (3), T82 (5), Y83 (6),	L79 (3)
N487	L24 (8), Y83 (4, **1**)	L24 (13), Y83 (5, **1**)
Y489	T27 (10), F28 (6), K31 (4), Y83 (1)	L24 (1), T27 (8), F28 (8), K31 (4), Y83 (1)
R493/Q493	K31 (4), S34 (8, **1**), E35 (4)	K31 (4, **1**), S34 (10, **1**), E35 (1). E38 (3)
S494		E38 (8, **2**)
Y495		E38 (2)
S496/G496	E38 (9), K353 (1)	E38 (1), K353 (6)
R498/Q498	E38 (8, **2**), H41 (1), Q42 (8, **1**), L45 (1)	H41 (8), L45 (4), K353 (1)
T500	H41 (1), L45 (2), N330 (5), D355 (8), R357 (4)	L45 (1), N330 (8), D355 (9, **2**), R357 (7)
Y501/N501	E38 (3), H41 (10), K353 (22), G354 (1)	H41 (5), K353 (9)
G502	K353 (3, **1**), G354 (7), D355 (1)	K353 (4, **1**), G354 (7), D355 (2)
H505/Y505	E37 (4), K353 (31), G354 (4)	E37 (3), K353 (25), G354 (5), R393 (1)
Total	244, **9**	234, **10**

The numbers in parentheses of eqACE2 (Omicron BA.1-RBD) and eqACE2 (SARS-COV-2 PT-RBD) residues represent the number of vdw contacts between the indicated residues with eqACE2. The numbers with underline suggest numbers of potential H-bonds between the pairs of residues. vdw contact was analyzed at a cutoff of 4.5 Å and H-bonds at a cutoff of 3.5 Å.

Supplementary Table 6 Amino acid comparison of hACE2 and eqACE2 interacting with Omicron BA.1-RBD

eqACE2/hACE2	Omicron BA.1-RBD (eqACE2)	Omicron BA.1-RBD (hACE2)
S19	N477 (5, **1**)	A475 (3, **1**), G476 (3), N477 (11, **1**)
L24/Q24	A475 (2), G476 (4), N487 (8)	A475 (3, **1**), G476 (3), N477 (11, **1**)
T27	F456 (5), Y473 (1), A475 (3), Y489 (10),	F456 (5), Y473 (1), A475 (2), Y489 (5)
F28	Y489 (6),	Y489 (9)
E30/D30	L455 (2), F456 (4),	L455 (1), F456 (1)
K31	L455 (1), F456 (3), Y489 (4), R493 (4),	L455 (1), F456 (4), Y489 (6), R493 (4),
S34/H34	Y453 (2), R493 (8, **1**)	Y453 (10, **2**), R493 (11), S494 (8)
E35	R493 (4)	R493 (6, **1**)
E37	H505 (4)	H505 (2)
E38/D38	Y449 (9, **1**), S496 (9), Y501 (3) R498 (8, **2**)	Y449 (7, **2**), S496 (6), R498 (5, **2**), Y501 (1)
H41/Y41	R498 (1), T500 (1), Y501 (10),	R498 (3), T500 (7, **1**), Y501 (15)
Q42	Y449 (1, **1**), R498 (8, **1**)	Y449 (2, **1**), R498 (7)
L45	R498 (1), T500 (2)	T500 (1)
L79	F486 (3),	F486 (2)
T82/M82	F486 (5),	F486 (7)
Y83	F486 (6), N487 (4, **1**), Y489 (1)	F486 (11), N487 (10, **1**), Y489 (1, **1**)
Q325	F374 (4)	
N330	T500 (5)	T500 (8)
K353	R403 (1), S496 (1), Y501 (22), G502 (3, **1**), H505 (31)	Y495 (1), S496 (2), Y501 (21), G502 (6, **1**), H505 (27)
G354	Y501 (1),G502 (7), H505 (4)	G502 (7), H505 (4)
D355	T500 (8), G502 (1)	T500 (7), G502 (1)
R357	T500 (4)	T500 (3)
Total	244, **9**	287, **15**

The numbers in parentheses of Omicron BA.1-RBD (eqACE2) and Omicron BA.1 -RBD (hACE2) residues represent the number of vdw contacts between the indicated residues with

Omicron BA.1-RBD. The numbers with underline suggest numbers of potential H-bonds between the pairs of residues. vdw contact was analyzed at a cutoff of 4.5 Å and H-bonds at a cutoff of 3.5 Å.
