# Supplementary material for: Binding and structural basis of equine ACE2 to RBDs from SARS-CoV, SARS-CoV-2 and related coronaviruses
Source: Nat Commun. 2022 Jun 21;13:3547. doi: 10.1038/s41467-022-31276-6 (PMC9210341; doi:10.1038/s41467-022-31276-6)
Supplement: Supplementary file 3 — Reporting Summary [file 41467_2022_31276_MOESM3_ESM.pdf]

nature portfolio

Corresponding author(s): Qihui Wang, Kefang Liu

Last updated by author(s): Jun 2, 2022

Reporting Summary

Nature Portfolio wishes to improve the reproducibility of the work that we publish. This form provides structure for consistency and transparency in reporting. For further information on Nature Portfolio policies, see our Editorial Policies and the Editorial Policy Checklist.

Statistics

For all statistical analyses, confirm that the following items are present in the figure legend, table legend, main text, or Methods section.

n/a	Confirmed	
☐	☒	The exact sample size (*n*) for each experimental group/condition, given as a discrete number and unit of measurement
☐	☒	A statement on whether measurements were taken from distinct samples or whether the same sample was measured repeatedly
☐	☒	The statistical test(s) used AND whether they are one- or two-sided<br>*Only common tests should be described solely by name; describe more complex techniques in the Methods section.*
☒	☐	A description of all covariates tested
☒	☐	A description of any assumptions or corrections, such as tests of normality and adjustment for multiple comparisons
☒	☐	A full description of the statistical parameters including central tendency (e.g. means) or other basic estimates (e.g. regression coefficient) AND variation (e.g. standard deviation) or associated estimates of uncertainty (e.g. confidence intervals)
☒	☐	For null hypothesis testing, the test statistic (e.g. *F*, *t*, *r*) with confidence intervals, effect sizes, degrees of freedom and *P* value noted<br>*Give P values as exact values whenever suitable.*
☒	☐	For Bayesian analysis, information on the choice of priors and Markov chain Monte Carlo settings
☒	☐	For hierarchical and complex designs, identification of the appropriate level for tests and full reporting of outcomes
☒	☐	Estimates of effect sizes (e.g. Cohen's *d*, Pearson's *r*), indicating how they were calculated

Our web collection on statistics for biologists contains articles on many of the points above.

Software and code

Policy information about availability of computer code

Data collection	Data of size exclusion were collected using UNICORN 7.5. Data of flow cytometry were collected using BD FACS Canto Diva 8.0.3 (BD Biosciences). Data of SPR analysis were collected using BIAcoreTM 8K control software 3.0.12.15655 (GE Healthcare).
Data analysis	The data of all flow cytometry samples were analyzed using FlowJo 7.6 (TreeStar Inc., Ashland, OR, USA). Binding kinetics of SPR analysis were analyzed with BiacoreTM Insight Evaluation software 3.0.12.15655 (GE healthcare). Diffraction data were processed using HKL2000 ver721. The structure of 2 complexes were determined by the molecular replacement method using Phaser 2.8. The atomic models were completed with Coot 0.9.4 and refined with Phenix 1.20.1, and the stereochemical qualities of the final models were assessed with MolProbity 4.5. The figures illustrating complex structures were drawn by PyMOL 2.4.1.

For manuscripts utilizing custom algorithms or software that are central to the research but not yet described in published literature, software must be made available to editors and reviewers. We strongly encourage code deposition in a community repository (e.g. GitHub). See the Nature Portfolio guidelines for submitting code & software for further information.

Data

Policy information about availability of data

All manuscripts must include a data availability statement. This statement should provide the following information, where applicable:

- Accession codes, unique identifiers, or web links for publicly available datasets
- A description of any restrictions on data availability
- For clinical datasets or third party data, please ensure that the statement adheres to our policy

The atomic coordinates for the crystal structures of the eqACE2/RaTG13-RBD and eqACE2/SARS-COV-2 PT-RBD complexes have been deposited in the Protein Data Bank (www.rcsb.org) (PDB: 7W6R for eqACE2/RaTG13-RBD, 7W6U for eqACE2/SARS-COV-2 PT-RBD and 7XBY for eqACE2/Omicron BA.1-RBD). Source data has been

provided with this paper.

Field-specific reporting

Please select the one below that is the best fit for your research. If you are not sure, read the appropriate sections before making your selection.

☒ Life sciences ☐ Behavioural & social sciences ☐ Ecological, evolutionary & environmental sciences

For a reference copy of the document with all sections, see nature.com/documents/nr-reporting-summary-flat.pdf

Life sciences study design

All studies must disclose on these points even when the disclosure is negative.

Sample size	All flow cytometry experiments were performed in duplicate for each experiment. In the SPR analysis the binding kinetics were measured three times. Example size for both experiments were determined based on common practice (Wang et al., 2020, Cell, PMID: 32275855). Other experiments do not involve sample size determination.
Data exclusions	No data were excluded
Replication	All flow cytometry experiments were performed three independent replicates and each experiment were performed two replicates. In the SPR analysis, the binding kinetics were measured three times. All the results were replicable. Other experiments are about protein expression, protein purification and structure determination, therefore needs no replication.
Randomization	The study mainly tested binding of RBDs of SARS-CoV, SARS-CoV-2 prototype and variants of concern, and their close relative bat-origin RaTG13 and pangolin-origin GX/P2V/2017 and GD/1/2019 using equine ACE2 as receptor and determined crystal structures of eqACE2/RaTG13-RBD and eqACE2/SARS-CoV-2-RBD which need not randomization. Other experiments involve no randomization
Blinding	The study mainly tested binding of RBDs of SARS-CoV, SARS-CoV-2 prototype and variants of concern and their close relative bat-origin RaTG13 and pangolin-origin GX/P2V/2017 and GD/1/2019 using equine ACE2 as receptor and determined crystal structures of eqACE2/RaTG13-RBD and eqACE2/SARS-CoV-2-RBD which need no blinding. Other experiments involve no blinding.

Reporting for specific materials, systems and methods

We require information from authors about some types of materials, experimental systems and methods used in many studies. Here, indicate whether each material, system or method listed is relevant to your study. If you are not sure if a list item applies to your research, read the appropriate section before selecting a response.

Materials & experimental systems

n/a	Involved in the study
☐	☒ Antibodies
☐	☒ Eukaryotic cell lines
☒	☐ Palaeontology and archaeology
☒	☐ Animals and other organisms
☒	☐ Human research participants
☒	☐ Clinical data
☒	☐ Dual use research of concern

Methods

n/a	Involved in the study
☒	☐ ChIP-seq
☐	☒ Flow cytometry
☒	☐ MRI-based neuroimaging

Antibodies

Antibodies used	Anti-His/APC (Miltenyi Biotec, Cat# 130-119-820), RRID: AB_2751870. CB6 (Shi R et al., 2020, Nature, PMID: 32454512)
Validation	Anti-His/APC: RRID: AB_2751870, relevant information can be found in the following website. https://www.miltenyibiotec.com/_Resources/Persistent/9eea51b579c367b02e27e31fd374864009b00ebb/DS_His_Antibody_APC_GG11-8F3.5.1_130-119-820.pdf<br>CB6: The antibody was reported by our lab and (Shi R et al., 2020, Nature, PMID: 32454512) and was expressed ourselves in this project (see Methods). The antibody has been commercialized (commercial name etesevimab) and relevant information can be seen in the following website: https://www.ncbi.nlm.nih.gov/books/NBK572103/

Eukaryotic cell lines

Policy information about cell lines

Cell line source(s)	BHK-21 cells (ATCC CCL-10), Expi293F cells were from Gibco (A14527)
Authentication	All the cells were authenticated with morphology.

Mycoplasma contamination	We confirm that all cell lines negative for mycoplasma contamination.
Commonly misidentified lines (See ICLAC register)	No misidentified cell lines were used.

Flow Cytometry

Plots

Confirm that:

- [x] The axis labels state the marker and fluorochrome used (e.g. CD4-FITC).
- [x] The axis scales are clearly visible. Include numbers along axes only for bottom left plot of group (a 'group' is an analysis of identical markers).
- [x] All plots are contour plots with outliers or pseudocolor plots.
- [x] A numerical value for number of cells or percentage (with statistics) is provided.

Methodology

Sample preparation	The plasmids containing hACE2 or eqACE2 fused with eGFP were transfected into BHK-21 cells. A mixture containing SARS-CoV-2-RBD (5 μg/mL) or RaTG13-RBD (5 μg/mL) and CB6 antibody were pre-incubated at 4 oC for 1h, and then incubated with the BHK cells at 4 oC for 1h. Subsequently, cells were washed with PBS thrice and stained with APC mouse anti-his secondary antibody for 1h
Instrument	BD FACS Canto FlowCytometer (BD Biosciences)
Software	Data was collected using FACS Diva 8.0.3 software. FlowJo 7.6 was used to analyse the flow cytometry data.
Cell population abundance	Each samples collected 10000 cells.
Gating strategy	How the boundary were defined were indicated in Fig. S5A. The boundary of positive and negative staining were defined based on the negative control cells which were not transfected with ACE2, thus express no GFP and can not be stained by RBDs.

- [x] Tick this box to confirm that a figure exemplifying the gating strategy is provided in the Supplementary Information.
